# Different types of electrostimulation target specific impaired sensory and motor functions in chemotherapy-induced peripheral neuropathy: a secondary analysis of a controlled trial

**DOI:** 10.3389/fneur.2026.1653161

**Published:** 2026-03-04

**Authors:** Florian Rieder, Robert Sassmann, Yvonne Theres Kienberger, Vanessa Castagnaviz, Dagmar Schaffler-Schaden, Tim Johansson, Gabriel Rinnerthaler, Maria Flamm, Richard Greil, Christoph Schulze, Simon Peter Gampenrieder

**Affiliations:** 1 Institute of Physical Medicine and Rehabilitation, Paracelsus Medical University, Salzburg, Austria; 2 IIIrd Medical Department with Haematology, Medical Oncology, Haemostaseology, Infectiology and Rheumatology, Oncologic Center, Paracelsus Medical University, Salzburg, Austria; 3 Salzburg Cancer Research Institute (SCRI) - Laboratory for Immunological and Molecular Cancer Research (LIMCR) and Center for Clinical Cancer and Immunology Trials (CCCIT), Salzburg, Austria; 4 Cancer Cluster Salzburg, Salzburg, Austria; 5 Institute of General Practice, Family Medicine and Preventive Medicine, Paracelsus Medical University, Salzburg, Austria; 6 Salzburg Regional Health Fund (SAGES), Salzburg, Austria; 7 Division of Clinical Oncology, Department of Internal Medicine, Medical University of Graz, Graz, Austria

**Keywords:** CIPN, electrotherapy, HTEMS, QLQ-CIPN20, TENS

## Abstract

**Background:**

We performed a secondary data analysis of a previously published study to investigate the effects of different electrical stimulation modalities on specific impaired sensory and motor functions.

**Methods:**

A total of 51 patients with chemotherapy-induced peripheral neuropathy (CIPN) ≥ grade 1 after receiving platinum- and/or taxane-based chemotherapy were randomized to 8 weeks of high-tone external muscle stimulation (HTEMS) or transcutaneous electrical nerve stimulation (TENS). A control group (*n* = 17) receiving no intervention was recruited retrospectively. Patients received 8 weeks of home-based electrotherapy for at least 5 days a week, for 30 min per day, using either a TENS or HTEMS device. In the original study, changes in the EORTC-QLQ-CIPN20 questionnaire were measured before and after the intervention. For this secondary data analysis, we performed sub-analyses to examine the specific effects of TENS and HTEMS on the individual sensory and motor scale outcomes of the EORTC-QLQ-CIPN20 questionnaire.

**Results:**

For sensory function categories, HTEMS significantly improved tingling in the fingers or hands (*p* = 0.009) and the numbness in the toes or feet (*p* = 0.018). TENS tended to reduce shooting or burning pain in the toes or feet (*p* = 0.051). TENS also demonstrated a trend to improve problems in standing or walking due to difficulties in feeling the ground (*p* = 0.051), while improvements after HTEMS reached significance (*p* = 0.045). For motor function categories, TENS improved difficulties opening a bottle due to weakness (*p* = 0.036), and HTEMS reduced difficulties in walking due to downward dropping of the feet (*p* = 0.015). There were no changes for any category in the control group.

**Conclusion:**

Electrotherapy is a useful tool for treating CIPN. A symptom-oriented selection of the stimulation modality may be promising.

**Clinical trial registration:**

https://clinicaltrials.gov/ct2/show/NCT03978585

## 1 Introduction

Cancer patients receiving neurotoxic chemotherapy often develop impaired sensory and motor functions, such as tingling, numbness, hypesthesia, and weakness in the hands, fingertips, feet, or toes (1). Chemotherapy-induced peripheral neuropathy (CIPN) is a serious side effect of anticancer therapies and is difficult to treat, as drugs and other conservative therapies often provide unsatisfying results (2). Although evidence is still limited, recent small randomized controlled trials have demonstrated that electrotherapy can effectively reduce CIPN symptoms to a clinically relevant extent (3, 4). High-tone external muscle stimulation (HTEMS) and transcutaneous electrical nerve stimulation (TENS) are the most frequently used methods (3–5). In a previously conducted randomized controlled trial (3), we were able to demonstrate that both electrical modalities increase impaired sensory and motor functions, as measured by the EORTC-QLQ-CIPN20 questionnaire. Improvements in the global scores did not differ between HTEMS and TENS. However, symptoms experienced by CIPN patients are very heterogeneous, and it is not clear how the different stimulation methods affect different complaints. Therefore, the effect of HTEMS and TENS on the individual questions of the EORTC-QLQ-CIPN20 questionnaire was explored in a secondary data analysis of our previously published study (3).

## 2 Materials and methods

This is a secondary explorative data analysis of a previously published randomized controlled clinical trial comparing 8 weeks of home-based HTEMS or TENS therapy to a retrospectively recruited control group. This study was registered at ClinicalTrials.gov^1^ and approved by the Ethics Committee of Salzburg County (ID 415-E/2376/7–2018). Reporting follows the CONSORT guidelines (see CONSORT checklist and study flow in the Supplementary material). A detailed description of the methods can be found in the studies by Sassmann and Gampenrieder (3) and Schaffler-Schaden and Sassmann (6).

### 2.1 Patients and treatment

Adult male and female patients who had completed chemotherapy with a taxane or platinum salt for confirmed invasive cancer (any entity) and had a clinical diagnosis of CIPN of ≥ grade 1 according to the Common Terminology Criteria for Adverse Events version 4 (CTCAE v 4) and an Eastern Cooperative Oncology Group (ECOG) performance score of 0–1 were randomized to either HTEMS or TENS therapy. Patient recruitment and randomization were conducted from September 2019 to October 2021. A control group (*n* = 17) was recruited retrospectively from July 2022 to March 2023 to better estimate the effects of electrotherapy. Patients randomized to the intervention performed 30 min of home-based electrotherapy daily, or at least 5 days a week, for 8 weeks on the feet and, if also affected, on the hands. Electrotherapy settings were set in accordance with the manufacturer’s recommendations for peripheral nerve pain. The exact intervention settings can be found in the Supplementary material. Patients in the control group did not receive any therapy for CIPN symptoms for a duration of 8 weeks but completed the EORTC-QLQ-CIPN20 questionnaire before and after this period.

### 2.2 Outcome measurements

All outcome measurements were performed before and after the 8 weeks of intervention. In the original study, the primary outcome parameters were changes in the EORTC-QLQ-CIPN20 questionnaire; secondary outcomes were improvements in the patient’s quality of life (EORTC-QLQ-C30), the classification of CIPN grade, and clinician-reported outcomes, including vibration sensibility, Achilles and patellar tendon reflexes, temperature sensibility, perception of touch, and strength of the lower leg muscles.

The EORTC-QLQ-CIPN20 contains 20 items assessing sensory (9 items), motor (8 items), and autonomic symptoms (3 items), using a 4-point Likert scale. For this secondary explorative data analysis, the questions for the sensory and motor impairments were evaluated separately and compared between the groups. The category of autonomic scales, which is known to show low reliability and validity (7), was excluded from the analysis.

### 2.3 Statistical analysis

Normal distribution of data was assessed using the Shapiro–Wilk test. Pre–post changes between the groups in the main categories of the EORTC-QLQ-CIPN20 questionnaire were analyzed using a Kruskal–Wallis test for non-normally distributed data. If a significant effect was found, the Mann–Whitney U-tests were performed *post-hoc* with Bonferroni-corrected *p*-values. Pre–post changes in the individual questions for sensory and motor impairments were analyzed within the groups using a Wilcoxon matched pairs test with Bonferroni-corrected p-values. All statistical analyses were performed using IBM SPSS Statistics 23.0 (IBM, Ehningen, Germany). Figures were created using GraphPad Prism v.9 (GraphPad Software, Boston, United States).

## 3 Results

A total of 51 patients were randomized to the TENS or HTEMS group. One patient in the HTEMS group died due to cancer during the study period and was not included in the analyses. Thus, 25 patients in the HTEMS group and 25 patients in the TENS group completed the EORTC-QLQ-CIPN20 questionnaires before and after the 8 weeks of intervention. Overall, 17 patients in the retrospectively recruited control group completed this questionnaire twice. Baseline characteristics of the patients are presented in Table 1. There were no baseline differences between the groups in the EORTC-QLQ-CIPN20 scores. Examining the individual questions, the groups differed on the items ‘cramps in the hands’ (*p* = 0.003) and ‘cramps in the feet’ (*p* = 0.011), with higher mean rank values for the control group in both categories (tested using the Kruskal–Wallis test). Baseline characteristics are also reported in Sassmann et al. and in the Supplementary material.

**Table 1:** Frequency of individual sensory and motor impairments and the successful electrotherapy device for each item.

Category	Item	Frequency (%): quite a bit + very much	HTEMS			TENS			CON		
			Median (Minimum–Maximum) T0	Median (Minimum–Maximum) T1	*p*-value (Bonferroni-corrected) effect size (r)	Median (Minimum–Maximum) T0	Median (Minimum–Maximum) T1	*p*-value (Bonferroni-corrected) effect size (r)	Median (Minimum–Maximum) T0	Median (Minimum–Maximum) T1	*p*-value (Bonferroni-corrected) effect size (r)
S	**Did you have numbness in your toes or feet?**	80.6	**4 (1–4)**	**3 (1–4)**	**0.018** ***r* = 0.389**	4 (1–4)	3 (1–4)	0.855 *r* = 0.151	3 (1–4)	3 (1–4)	>1.0 *r* = 0.631
S	Did you have tingling toes or feet?	74.7	4(1–4)	3(1–4)	0.09 *r* = 0.306	3(1–4)	3(1–4)	0.63 *r* = 0.177	3(1–4)	3(1–4)	>1 *r* = 0.083
S	**Did you have tingling fingers or hands?**	64.2	**4 (1–4)**	**3 (1–4)**	**0.009** ***r* = 0.417**	3 (1–4)	3 (1–4)	0.117 *r* = 0.292	3 (1–4)	3 (1–4)	>1 *r* = 0.000
S	Did you have numbness in your fingers or hands?	59.7	3 (1–4)	2 (1–4)	0.435 *r* = 0.206	3 (1–4)	3 (1–4)	0.192 *r* = 0.262	3 (1–4)	2 (1–4)	0.288 *r* = 0.286
M	Did you have difficulty manipulating small objects with your fingers?	53.8	2 (1–4)	2 (1–4)	>1 *r* = 0.130	3 (1–4)	3 (1–4)	0.21 *r* = 0.256	2 (1–4)	2 (1–4)	>1 *r* = 0.171
M	**Did you have difficulty opening a jar or a bottle because of weakness in your hands?**	47.7	2 (1–4)	2 (1–4)	0.087 *r* = 0.309	**3 (1–4)**	**2 (1–4)**	**0.036** ***r* = 0.353**	2 (1–4)	2 (1–4)	0.177 *r* = 0.324
S	**Did you have shooting or burning pain in your toes or feet?**	43.3	1 (1–4)	1 (1–3)	0.09 *r* = 0.307	**3 (1–4)**	**1 (1–4)**	**0.051** ***r* = 0.338**	1 (1–4)	1 (1–3)	>1 *r* = 0.165
S	**Did you have problems standing or walking because of difficulty feeling the ground under your feet?**	32.8	**2 (1–4)**	**1 (1–4)**	**0.045** ***r* = 0.344**	**2 (1–4)**	**1 (1–4)**	**0.051** ***r* = 0.338**	2 (1–4)	2 (1–4)	>1 *r* = 0.140
M	Did you have difficulty climbing stairs or getting up out of a chair because of weakness in your legs?	26.8	1 (1–4)	1 (1–4)	>1 *r* = 0.098	2 (1–4)	2 (1–4)	0.396 *r* = 0.213	2 (1–4)	2 (1–3)	>1 *r* = 0.052
S	Did you have shooting or burning pain in your fingers or hands?	25.4	1 (1–4)	1 (1–4)	0.489 *r* = 0.197	1 (1–4)	1 (1–4)	0.102 *r* = 0.300	1 (1–3)	1 (1–3)	0.702 *r* = 0.204
M	Did you have a problem holding a pen, which made writing difficult?	22.4	1 (1–4)	1 (1–4)	0.471 *r* = 0.200	2 (1–4)	1 (1–4)	0.174 *r* = 0.268	1 (1–4)	1 (1–4)	0.951 *r* = 0.171
S	Did you have difficulty distinguishing between hot and cold water?	17.9	1 (1–4)	1 (1–4)	0.06 *r =* 0.330	1 (1–4)	1 (1–4)	>1 *r =* 0.106	1 (1–3)	1 (1–3)	>1 *r =* 0.000
M	Did you have cramps in your feet?	16.5	1 (1–4)	1 (1–2)	0.891 *r* = 0.148	1 (1–4)	1 (1–4)	0.663 *r* = 0.173	2 (1–4)	2 (1–3)	>1 *r* = 0.077
M	**Did you have difficulty walking because your feet dropped downward?**	12	**1 (1–4)**	**1 (1–3)**	**0.015** ***r* = 0.397**	1 (1–4)	1 (1–2)	>1 *r* = 0.115	1 (1–3)	1 (1–2)	>1 *r* = 0.000
S	Did you have difficulty hearing?	9	1 (1–4)	1 (1–4)	0.618 *r* = 0.179	1 (1–3)	1 (1–3)	0.771 *r* = 0.160	1 (1–4)	1 (1–3)	>1 *r* = 0.000
M	Did you have cramps in your hands?	4.5	1 (1–2)	1 (1–2)	>1 *r* = 0.000	1 (1–2)	1 (1–2)	0.540 *r* = 0.190	1 (1–4)	1 (1–3)	>1 *r* = 0.121

M = question in the motor scale, S = question in the sensory scale, 1 = not at all, 2 = a little, 3 = quite a bit, 4 = very much, the frequency reflects the number of patients who answered “quite a bit” and “very much” in the questionnaire. HTEMS, high-tone external muscle stimulation; TENS, transcutaneous electrical nerve stimulation; CON, control group, bold letters = electrical intervention led to a significant reduction of symptom severity, T0 = baseline, T1 = follow-up, effect size (r): 0.00–0.300 = small; 0.301–0.500 = moderate; > 0.500 = large; calculated as Z value / $\sqrt{n}$ .

Overall scores for the sensory and motor impairment scales decreased significantly in the HTEMS (sensory: from 45.0 ± 21.2 to 32.7 ± 15.4, *p* < 0.001; motor: from 32.4 ± 18.2 to 24.2 ± 15.7, *p* = 0.045) and TENS groups (sensory: from 47.3 ± 17.5 to 32.6 ± 17.7, *p* = 0.018; motor: from 25.9 ± 20.6 to 17.7 ± 17.3, *p* = 0.036) but not in the control group (sensory: from 36.4 ± 21.7 to 33.2 ± 22.4, *p* = 0.882; motor: from 29.4 ± 22.7 to 26.6 ± 24.7, *p* = 0.079). There were no baseline differences between the groups.

**Figure 1:**
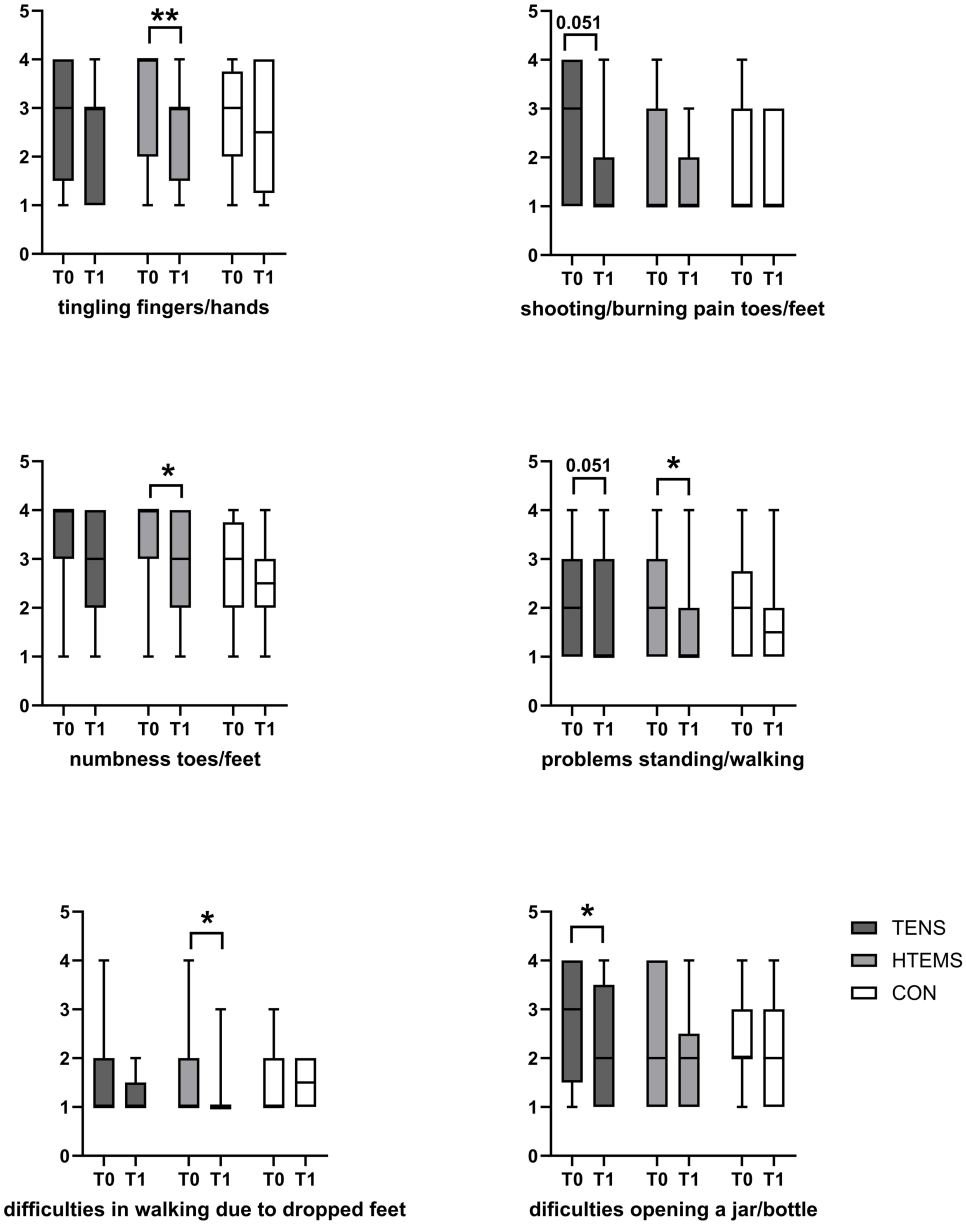
Symptom reduction after electrotherapy in items of the EORTC QLQ-CIPN20 questionnaire, 1 = not at all, 2 = a little, 3 = quite a bit, 4 = very much. TENS, transcutaneous electrical nerve stimulation; HTEMS, high-tone external muscle stimulation; CON, control group; *significant differences *p* < 0.05, **significant differences *p* < 0.01, data are presented as boxplots (median, minimum, and maximum).

Looking at the individual questions for sensory impairments, HTEMS improved tingling in the fingers or hands (*p* = 0.009) and the numbness in the toes or feet (*p* = 0.018; Figure 1; Table 1). Tingling of the feet or toes also decreased after HTEMS but did not reach statistical significance (*p* = 0.09). HTEMS also improved patients’ ability to stand or walk without problems related to impaired sensation (*p* = 0.045), whereas TENS only showed a trend toward improvement (*p* = 0.051; Figure 1; Table 1). TENS also showed a trend toward reducing shooting or burning pain in the toes or feet (*p* = 0.051). For motor function questions, TENS improved difficulties opening a bottle due to weakness (*p* = 0.036), while HTEMS reduced difficulties in walking caused by the downward dropping of the feet (*p* = 0.015). No changes were observed for the other questions (Figure 1; Table 1), and the control group showed no significant changes for any question. All reported *p*-values were Bonferroni-corrected.

## 4 Discussion

The improvements observed in impaired sensory and motor functions after electrotherapy, measured globally using the EORTC-QLQ-CIPN20 questionnaire, were clinically meaningful with no significant differences between the two techniques. This is a step forward in the treatment of CIPN since previous strategies were less successful (2, 8, 9). Additionally, TENS and HTEMS treatments can be easily performed alone at home and show no relevant side effects (3).

However, individual sensory and motor problems that affect patients during activities of daily living can be highly heterogeneous and may not be sufficiently represented by a global score. In our population, the most frequently reported problems, consistent with previous studies (10, 11), were tingling and numbness in the toes or feet and in the hands or fingers, difficulty opening a bottle due to weakness in the hands, and shooting or burning pain in the toes or feet. After 8 weeks of electrotherapy, our results showed improvements in the majority of these items, although the magnitude of improvement varied depending on the stimulation modality. HTEMS appeared to especially target tingling and burning sensations, whereas TENS mainly reduces pain. For impairments in walking or performing tasks such as opening objects, the results were less clear. It is possible that both numbness and pain in the feet impair standing and walking, and alleviating even one of these symptoms could lead to improvements in this category.

### 4.1 Limitations

When interpreting the results of this study, some limitations should be considered. The retrospective nature of the control group is a major limitation of our study. The allocation of patients to this group was not randomized and was carried out 7 months after the end of the original study. Selection biases and time-related confounding factors (e.g., changes in chemotherapy regimens) may have influenced the results. Furthermore, despite the significant improvements in the individual items of the EORTC-QLQ-CIPN20 questionnaire, it is not known whether these are clinically relevant, as no values for minimally clinically relevant changes for these items have yet been established. In addition, *a priori* sample size calculation was only performed for the global EORTC-QLQ-CIPN 20 scores. The individual item analyses can therefore be slightly underpowered. Finally, the small sample size of this study did not allow us to investigate the influence of covariables such as sex, age, and BMI on the outcomes of TENS or HTEMS therapy, nor were comorbidities systematically recorded. Therefore, future multicenter studies using a large sample size are needed to answer these questions.

## 5 Conclusion

Electrotherapy appears to be an effective tool for alleviating impaired sensory and motor functions in CIPN patients. Furthermore, this secondary exploratory data analysis suggests that HTEMS specifically targets tingling and burning sensations, while TENS appears to primarily relieve pain. However, this needs to be confirmed by further well-powered clinical studies before practicing physicians can prescribe symptom-oriented electrotherapy.

## Data Availability

The raw data supporting the conclusions of this article will be made available by the authors, without undue reservation.

## Footnotes

1
https://clinicaltrials.gov/ct2/show/NCT03978585
