# Supplementary material for: Different types of electrostimulation target specific impaired sensory and motor functions in chemotherapy-induced peripheral neuropathy: a secondary analysis of a controlled trial
Source: Front Neurol. 2026 Mar 4;17:1653161. doi: 10.3389/fneur.2026.1653161 (PMC12995806; doi:10.3389/fneur.2026.1653161)
Supplement: Supplementary file 1 [file Table_1.DOCX]

**Sudy Flow**


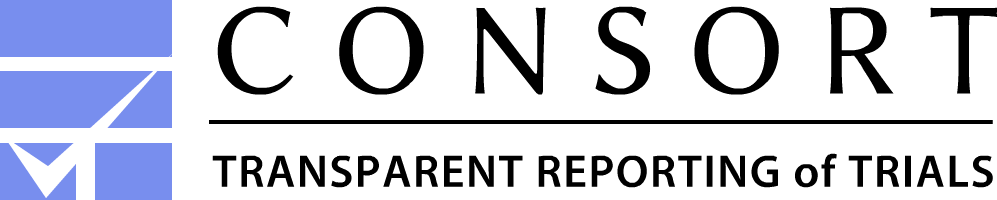


**CONSORT 2010 Flow Diagram**

## Follow-Up

Lost to follow-up (n=1) deceased

## Allocation

Excluded (n=2)

♦ Not meeting inclusion criteria (n=2)

Analysed (n=25)

Assessed for eligibility (n=53)

## Enrollment

to intervention HTEMS (n=26)

♦ Received allocated intervention (n=26)

to intervention TENS (n=25)

♦ Received allocated intervention (n=25)

Randomized (n=51)

Analysed (n=25)

## Analysis

Analysed (n =17)

Lost to follow up (n=5)

CON (n=26)

♦ excluded (n=3) not meeting inclusion criteria

♦ ongoing neurotoxic chemotherapy (n=1)

recruited in retrospect

**Baseline characteristics of patients**

|  | **HTEMS**  n = 25 | **TENS**  n = 25 | **Controls**  n = 17 | **p-value** |
| --- | --- | --- | --- | --- |
| **Median age [years] (range)** | 63 (36-89) | 69 (24-81) | 63 (47-79) | 0.748 |
| **Age <60y** | 6 (24%) | 11 (44%) | 4 (23%) |  |
| **Gender** |  |  |  | 0.832 |
| Male | 10 (40%) | 12 (48%) | 10 (59%) |  |
| female | 15 (60%) | 13 (52%) | 7 (41%) |  |
| **Tumor entity** |  |  |  | 0.922 |
| Breast cancer | 11 (44%) | 10 (40%) | 5 (29%) |  |
| Colorectal cancer | 4 (16%) | 6 (24%) | 5 (29%) |  |
| Esophageal cancer | 2 (8%) | 3 (12%) | 2 (12%) |  |
| Pancreatic cancer | 1 (4%) | 4 (16%) | 2 (12%) |  |
| Gastric cancer | 2 (8%) | 2 (8%) | 2 (12%) |  |
| Other | 5 (20%) | 0 (0%) | 1 (6%) |  |
| **Tumor AJCC stage** |  |  |  |  |
| I | 3 (12%) | 3 (12%) | 2 (12%) | 0.543 |
| II | 6 (24%) | 8 (32%) | 4 (23%) |  |
| III | 8 (32%) | 2 (8%) | 5 (29%) |  |
| IV | 8 (32%) | 12 (48%) | 6 (35%) |  |
| **Grade** |  |  |  | 0.910 |
| 1-2 | 12 (48%) | 11 (44%) | 6 (35%) |  |
| 3 | 7 (28%) | 10 (40%) | 6 (35%) |  |
| Unknown | 6 (24%) | 4 (16%) | 5 (30) |  |
| **Therapeutic setting** |  |  |  |  |
| Curative | 16 (64%) | 15 (60%) | 11 (65%) | 0.939 |
| Palliative | 9 (36%) | 10 (40%) | 6 (35%) |  |
| **Neurotoxic chemotherapy*** |  |  |  | 0.049 |
| Taxane | 12 (48%) | 11 (44%) | 6 (35%) |  |
| Platinum^#^ | 8 (32%) | 9 (36%) | 8 (47%)^#^ |  |
| Taxane and Platinum | 5 (20%) | 5 (20%) | 3 (18%) |  |
| **Mean duration of neurotoxic chemotherapy [days] (SD)** | 104 (34) | 134 (101) | 143 (72) | 0.193 |
| Median duration [days] (range) | 105 (140) | 89 (477) | 170 (270) |  |
| Early discontinuation because of CIPN | 10 (40%) | 8 (32%) | 3 (18%) | 0.308 |
| **Mean time after ending of neurotoxic chemotherapy [days] (SD)** | 87(32) | 98 (39) | 97 (31) | 0.473 |
| **Dose reduction of neurotoxic chemotherapy** |  |  |  | 0.193 |
| Yes | 8 (32%) | 10 (40%) | 6 (35%) |  |
| No | 17 (68%) | 15 (60%) | 11 (65%) |  |
| **BMI at baseline** |  |  |  |  |
| Median (range) | 24.2 (19-36) | 25.8 (19-35) | 23.7 (17-31) | 0.215 |
| < 20 | 1 (4%) | 2 (8%) | 2 (12%) |  |
| 20-25 | 14 (56%) | 15 (60%) | 10 (59%) |  |
| > 25 | 10 (40%) | 8 (32%) | 5 (29%) |  |
| **Variation of BMI during neurotoxic chemotherapy [mean] (SD)** | -1.9 (7.1) | -2.0 (6.7) | -2,8 (7.3) | 0.230 |
| increase | 10 (40%) | 11 (44%) | 5 (30%) |  |
| loss ≤ 10% | 12 (48%) | 10 (40%) | 8 (47%) |  |
| loss of ≥ 10% | 3 (12%) | 4 (16%) | 4 (23%) |  |
| **Known Diabetes/Pre-Diabetes (y/n)** |  |  |  | 0.180 |
| Diabetes | 6 (24%) | 4 (16%) | 7 (41%) |  |
| No diabetes | 19 (76%) | 21 (84%) | 10 (59%) |  |
| **Therapy during electrotherapy** |  |  |  |  |
| Yes | 15 (60%) | 14 (56%) | 13 (77%) |  |
| No | 10 (40%) | 11 (44%) | 4 (23%) |  |
| Chemotherapy† | 6 (24%) | 2 (8%) | 7 (41%) |  |
| Chemotherapy† and targeted therapy or immunotherapy | 4 (16%) | 4 (16%) | 3 (18%) |  |
| Endocrine therapy | 2 (8%) | 2 (8%) | 1 (6%) |  |
| Targeted therapy ± endocrine therapy | 3 (12%) | 6 (24%) | 2 (12%) |  |
|  |  |  |  |  |

AJCC = American Joint Committee on Cancer; BMI = body mass index; HTEMS = high tone external muscle stimulation; TENS = transcutaneous electrical nerve stimulation; SD = standard deviation; * stratification factor for both intervention groups; # significant baseline differences between the control and the intervention groups; † non-neurotoxic chemotherapies only were allowed(e.g. capecitabine, 5-fluorouracil, irinotecan etc.)
